# Gender-Specific Effects of Depression and Suicidal Ideation in Prosocial Behaviors

**DOI:** 10.1371/journal.pone.0108733

**Published:** 2014-09-26

**Authors:** Ricardo Cáceda, Tori Moskovciak, Stefania Prendes-Alvarez, Justyna Wojas, Anzhelika Engel, Samantha H. Wilker, Jorge L. Gamboa, Zachary N. Stowe

**Affiliations:** 1 Psychiatric Research Institute, University of Arkansas for Medical Sciences, Little Rock, Arkansas, United States of America; 2 Department of Psychiatry and Behavioral Sciences, University of Miami Miller School of Medicine, Miami, Florida, United States of America; 3 Department of Medicine, Vanderbilt University, Nashville, Tennessee, United States of America; George Mason University/Krasnow Institute for Advanced Study, United States of America

## Abstract

**Background:**

Prosocial behaviors are essential to the ability to relate to others. Women typically display greater prosocial behavior than men. The impact of depression on prosocial behaviors and how gender interacts with those effects are not fully understood. We explored the role of gender in the potential effects of depression on prosocial behavior.

**Methods:**

We examined prosocial behaviors using a modified version of the Trust Game in a clinical population and community controls. Study participants were characterized on the severity of depression and anxiety, presence of suicidal ideation, history of childhood trauma, recent stressful life events, and impulsivity. We correlated behavioral outcomes with gender and clinical variables using analysis of variance and multiple regression analysis.

**Results:**

The 89 participants comprised four study groups: depressed women, depressed men, healthy women and healthy men (n = 16–36). Depressed men exhibited reciprocity more frequently than healthy men. Depression induced an inversion of the gender-specific pattern of self-centered behavior. Suicidal ideation was associated with increased reciprocity behavior in both genders, and enhancement of the effect of depression on gender-specific self-centered behavior.

**Conclusions:**

Depression, particularly suicidal ideation, is associated with reversal of gender-specific patterns of prosocial behavior, suggesting abnormalities in sexual hormones regulation. This explanation is supported by known abnormalities in the hypothalamus-pituitary-adrenal and hypothalamus-pituitary-gonadal axes found in depression.

## Introduction

Prosocial behaviors, defined as voluntary behaviors intended to benefit others, are central to the formation and maintenance of healthy interpersonal relationships, and for integration into society [1]. They are viewed as virtues in many cultures and include altruism, reciprocity and cooperation. Gender differences in prosocial behaviors have been well characterized. For example, women display greater and more frequent generosity and altruistic behavior than men [2]–[5]. Growing evidence supports that these gender differences in behavior are linked to sex hormones [6], [7].

Depression affects 10–15% of adults in the US and 350 million worldwide [8]. Patients with major depression frequently experience impaired social functioning, defined as an individual’s ability to perform and fulfill normal social roles [9]. The interaction between depression and prosocial behavior is unclear, at least partly because of the methodological challenge to establish causality and its directionality. Prosociality has been suggested to promote healthy development when appropriately regulated, but to increase the risk for psychopathology when overly low or high [10]. Whereas low prosocial behavior is associated with antisocial traits [11], high prosocial orientation may lead to over concern for others and high anxiety [12]. For instance, the differential gender rates for depression have been associated with preadolescent differences in prosociality associated with empathy and compliance [13]. Depression has also been associated with increased altruism and decreased self-centeredness, which can be explained by feelings of worthlessness, anxiety, and submissive behavior [14], leading to situations in which the depressed person is taken advantage of by others. On the other hand, depression can be seen as a state of increased anhedonia and self-centeredness [15], in which a person is unable to love him or herself and, as corollary, unable to care for somebody else. In contrast, it has been proposed that prosocial behaviors play a protective role against mood and anxiety disorders [16], [17]. Likewise, suicide has been seen as the product of both altruistic impetus (i.e., to spare my family or for the greater good of the community), and egoistic motivation (associated with low social integration) [18].

The purpose of the current study is to examine the impact of depression on prosocial behavior using a well validated behavioral economic tool: the Trust Game [19], [20], which involves a sequential economic exchange between two parties. We sought to extend previous investigations of depression by interrogating the impact of gender on the differences in prosocial behavior in depressed patients. Given the interaction of depression and hypothalamus function and the evidence that gonadal hormones mediate prosocial behaviors, we hypothesized that gender has an influence on the expression of prosocial behavior, which is reversed in depressed subjects.

## Methods

### Participants

We recruited four groups of adult (20–60 years old) volunteers: healthy women, healthy men, depressed women, and depressed men (See Table 1). Inclusion criteria for the depressed patient group were: a) meeting DSM-IV criteria for Major Depressive Disorder; and b) ability to provide written informed consent. Exclusion criteria were: a) history of schizophrenia, schizoaffective disorder or bipolar disorder; b) active substance use disorders, other than tobacco, by self-report; and c) suicide attempts in the last year. Depressed patients were recruited from the University of Miami and Jackson Memorial Hospital psychiatric outpatient clinics. Healthy participants were recruited from the Miami community.

**Table 1:** Demographic and clinical characteristics of depressed patients and healthy participants.

	Healthy women	Depressed women	Healthy men	Depressed men	F	P
N	16	36	17	20		
Age (years)	29.9±2.3	34.0±2.1	33.8±2.9	41.2±3.1	1.31	0.28
Education (years)	16.1±0.6	13.9±0.4^c^	16.1±0.9^d^^,^ b	13.5±1.3^c^	2.2	0.09
Student or working/unemployed,disabled or retired	12/0	15/14	10/1	6/8	11.06	<0.001*
Receiving antidepressant medication	0	24 (67%)	0	15 (75%)	32	<0.0001*
BDI-2	4.9±0.5^b^^,^ d	27.0±2.0^a^^,^ c	2.7±0.6^b^^,^ d	27.7±3.6^a^^,^ c	26.33	<0.001
Suicidal ideation	0	17	0	11	33.0	<0.0001*
Childhood trauma	33.2±4.2	45.5±4.3^c^	28.8±3.5^b^	34.9±6.6	2.02	0.118
Socio cultural stress	16.3±1.9^b^^,^ d	34.5±6.4^a^^,^ c	18.1±3.5^b^^,^ d	26.4±2.7^a^^,^ c	3.30	0.036
Social acceptability stress	8.0±0.5^b^^,^ d	12.7±0.7^a^^,^ c	7.6±0.6^b^^,^ d	12.3±1.3^a^^,^ c	8.60	<0.001
Social victimization stress	5.6±0.5^b^^,^ d	9.0±0.6^a^^,^ c	5.3±0.5^b^^,^ d	9.4±0.9^a^^,^ c	7.75	0.001
Work stress	10.0±0.6^b^	14.9±0.9^a^	10.6±0.8	13.4±1.2	4.57	0.016
Time pressure stress	18.0±1.4	19.7±1.0	15.2±2.0	18.8±1.8	0.85	0.513
Finances stress	10.5±1.6	14.4±1.0	8.5±1.0	14.6±1.0	3.57	0.037
Attentional impulsivity	14.6±0.8	19.1±0.9^c^	15.3±1.7^b^	17.3±0.9	3.41	0.032
Motor impulsivity	23.4±1.1	23.8±1.0	20.6±1.1	23.9±1.7	1.03	0.448
Nonplanning impulsivity	16.6±1.3	26.7±2.4^c^	16.6±1.2^b^^,^ d	26.7±2.4^c^	6.33	0.002

Bonferroni correction was used for multiple comparison;

a compared to suicide attempter group;

b compared to suicidal ideation group;

c compared to depressed control group;

d compared to healthy control group;

*Yates chi square.

### Ethics Statement

All procedures were approved by the University of Miami and Jackson Memorial Hospital Institutional Review Boards. Participants were remunerated for their participation.

### Procedures

After written informed consent, participants’ demographics, psychiatric history, and medical history were obtained. Next, participants completed the Beck Depression Inventory (BDI-2) [21], Beck Anxiety Inventory (BAI) [22], Columbia Suicide Severity Rating Scale (C-SSRS) [23], Barratt Impulsiveness Scale (BIS-11) [24], Childhood Trauma Questionnaire (CTQ) [25], and the Survey for Recent Life Experiences (SRLE) [26]. We measure suicidal ideation (SI) with the severity of suicidal ideation subscale of the C-SSRS applied to “currently” in order to identify current suicidal ideation. Following completion of these scales study participants were trained and then participated in a modified version of the Trust Game.

**Figure 1:**
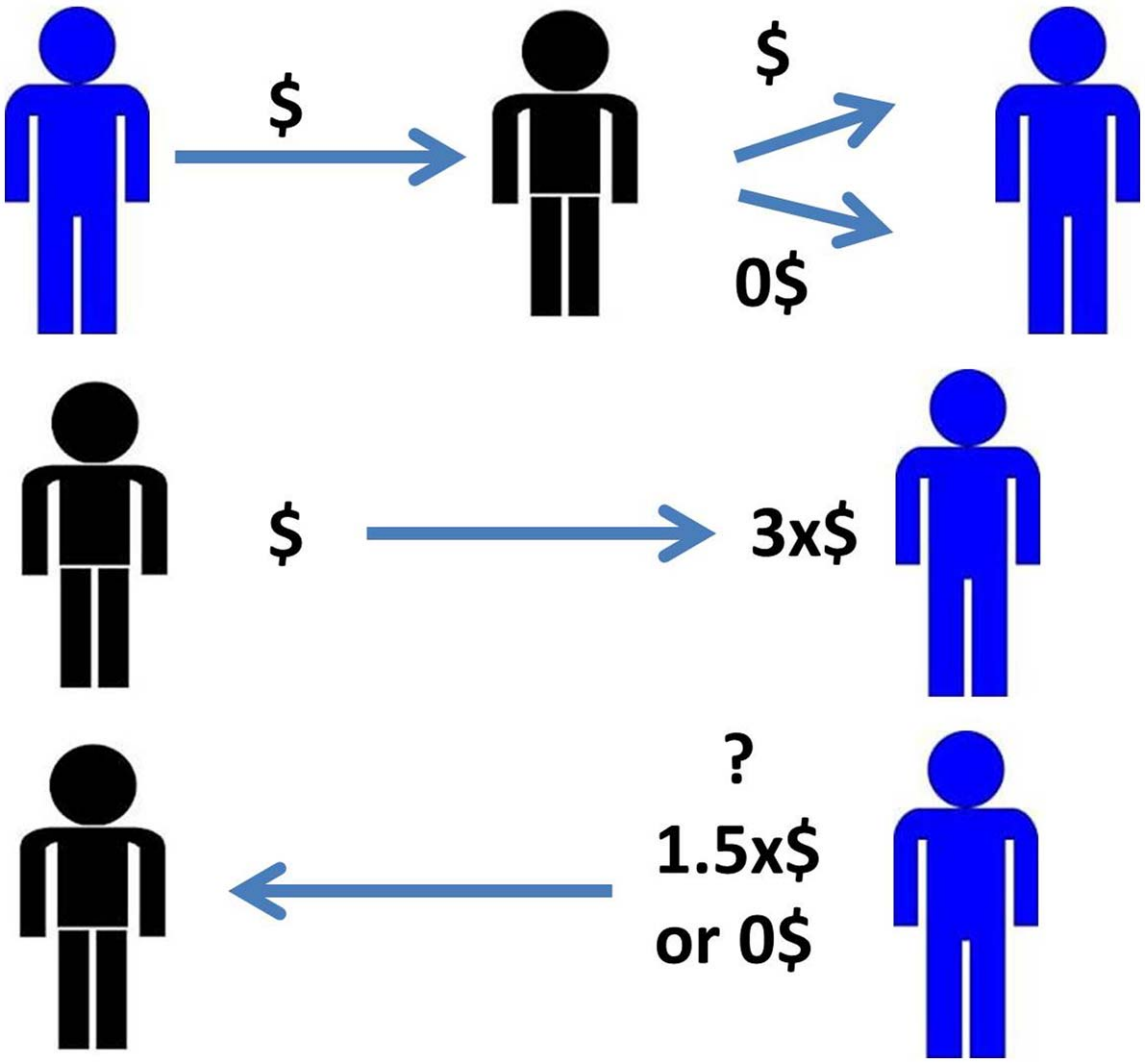
Depiction of the variations of the trust game. A. Baseline condition, the participant plays the role of trustee (blue) who receives a certain amount of money from the investor (3X) and needs to decide whether to give back some (1.5X) or none; B. Emotionally challenging condition, the participant plays two rounds with the same player. In the first, he/she plays the role of the investor who receives some money back only in half of the rounds; immediately afterwards roles are switched and the participant plays the role of trustee who must decide how much money to return.

*The Trust Game* is a standard two-person reciprocal exchange game widely employed to model interpersonal trust and willingness to reciprocate trust [19]. We used two computer-based trials in which each involved 30 rounds of one-time interactions between each study participant and different anonymous individuals. In the first trial or baseline condition, the participant (trustee role) and the other anonymous individual (the investor) were endowed with an initial pot of money ($X), then the investor sent a certain amount of money ($X) to the participant. Next, the participant received three times what the investor sent ($3X), and was asked to choose how much money he/she wanted to give back to the investor, either $1.5X or $0. During the second trial or “emotionally challenging condition,” the participant first played the role of the investor, and by design, in 50% of the rounds received some money back from the other player (trustee). Next, the roles were reversed with the participant playing the role of the trustee. (See Figure 1) The goal of the emotionally challenging condition was to expose a participant to either a fair or unfair experience, aiming to elicit an emotional response and detect its influence on the participant’s subsequent choice as trustee. In our study, reciprocity was operationally measured by the number of times participants gave money back in the first trial or baseline condition (expressed as a percentage of the total 30 rounds). This behavior has also been described as trustworthiness [4]. In the emotionally-challenging condition altruism was defined as the number of times participants gave money back despite not receiving any in the first round (expressed as a percentage of the number of times not receiving money back in the first round). Degree of self-centered behavior was measured by the number of times participants did not give money back on the second round after receiving money (expressed as a percentage of the number of times receiving money back in the first round).

### Data analysis

We used a two-way analysis of variance (ANOVA) to examine the main effects of either depression (depressed or healthy controls) or suicidal ideation (present or absent) status and gender and their interaction, and clinical measures and outcomes of the Trust Game as dependent variables. We also created dummy variables (healthy/non-suicidal females, healthy/non-suicidal males, depressed/suicidal females, and depressed/suicidal males) to compare differences among these four groups using ANOVA and Bonferroni correction for multiple comparisons. We also evaluated the effect of these four different groups and the offer amount on reciprocity behavior using a generalized lineal model. Model selection was based in the lower Akaike’s information criterion (AIC).

To examine individual variability of prosocial behavior with gender, depression, and other clinical variables we performed linear regression using reciprocity behavior in the baseline condition and self-centered behavior in the emotionally challenging condition as dependent variables, and depression severity, antidepressant use, presence of SI, anxiety, and recent social acceptability stress as independent variables. When a multiple regression was performed with these three variables it was non-significant.

Significance level was set at P≤0.05. We reported corrected P values [27].

## Results

Composition of the four study groups (healthy women, healthy men, depressed women, and depressed men) did not differ in age, racial composition or educational years. Depression severity (F(3,89) = 26.33, df = 86, P<0.001), presence of SI (Yates chi-squared test, *X^2^* = 37.13, P<0.0001), recent sociocultural stress (F(3,89) = 3.30, df = 85, P = 0.024), social acceptability stress (F(3,89) = 8.60, df = 86, P<0.001), and social victimization (F(3,89) = 7.75, df = 85, P = 0.001) were higher among the depressed groups than in the healthy control groups. (See Table 1).

### Baseline condition

**Figure 2:**
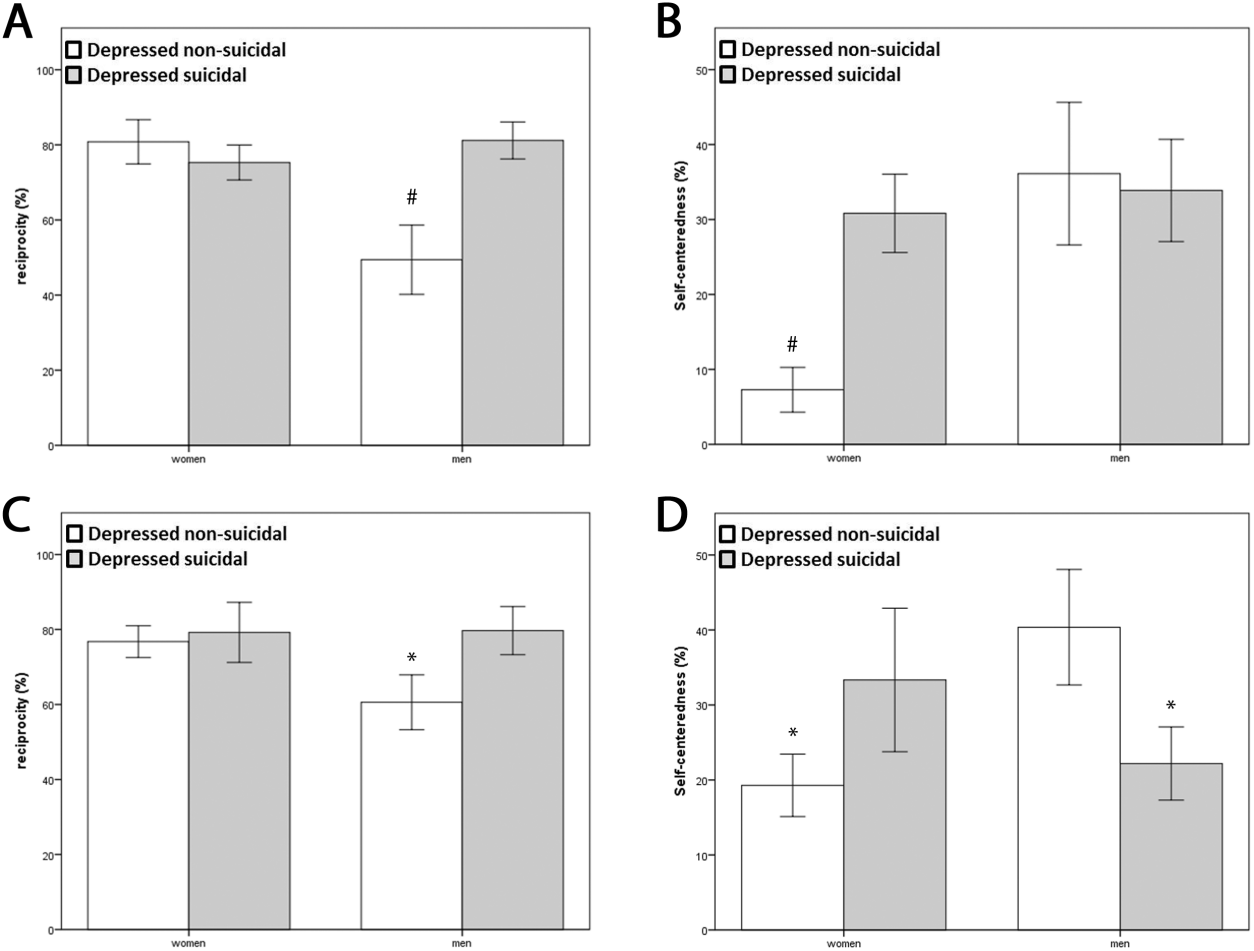
Effect of depression and presence of suicidal ideation in prosocial behavior in men and women. A. Reciprocity behavior during baseline condition in healthy participants and depressed patients. B. Self-centered behavior during emotionally challenging condition in healthy participants and depressed patients. C. Reciprocity behavior during baseline condition in depressed non suicidal and depressed suicidal patients. D. Self-centered behavior during emotionally challenging condition in in depressed non suicidal and depressed suicidal patients. * Different between healthy and depressed participants, # different from all other groups; p<0.05.

In the baseline condition, the main effect of depression (F(3,89) = 5.81, df = 86, P<0.001) and the interaction of gender × depression (P = 0.035) were significant. *Post-hoc* analysis revealed that healthy men displayed reciprocity behavior less frequently than depressed men and both women groups (Figure 2A). There was no SI in the healthy control groups. There was a significant main effect of SI on depressed patients’ reciprocity behavior (F(3,56) = 5.6, df = 54, P = 0.002). *Post-hoc* analysis revealed that reciprocity behavior was higher in men with SI than in those without SI (See Table 2 and Figure 2B).

**Table 2:** Behavior of depressed patients and healthy participants in the modified Trust Game.

						P		
	Healthy women^(a)^	Depressed women^(b)^	Healthy men^(c)^	Depressed men^(d)^	F	Gender effect	Depression effect	Gender × depression interaction
N	16	36	17	20				
Reciprocity	80.4±6.2^c^	75.6±4.5^c^	46.4±9.3^a^^,^ b ^,^ d	81.2±4.9^c^	3.68	0.178	0.073	0.026
Altruistic behavior	62.5±9.4	56.1±5.0	46.3±9.6	52.3±7.4	0.34	0.223	0.622	0.371
Self-centered behavior	7.2±3.2^b^^,^ c ^,^ d	30.2±5.1^a^	39.6±9.8^a^	21.4±4.0^a^	2.45	0.085	0.042	0.201

Bonferroni correction was used for multiple comparison;

a compared to suicide attempter group;

b compared to suicidal ideation group;

c compared to depressed control group;

d compared to healthy control group.

### Emotionally challenging condition

In the emotionally challenging condition, no difference was observed between the four groups in altruistic behavior. For self-centered behavior (not giving after receiving), the main effect of gender (F(3,89) = 3.47, df = 86, P = 0.02) and the interaction of gender × depression was significant (P = 0.001). *Post-hoc* analysis revealed that healthy women exhibited less frequent self-centered behavior compared to all the other groups (See Table 2 and Figure 2C).

Closer scrutiny of the effects of SI on prosocial behavior revealed no effect on altruistic behavior. Regarding self-centered behavior, we observed a significant interaction of gender × SI (F(3,56) = 7.38, df = 54, P<0.001). *Post-hoc* analysis revealed that women with SI exhibited more frequent self-centered behavior than women without SI. However, men showed the opposite pattern: suicidal men displayed less self-centered behavior compared to non-suicidal men (Figure 2D).

### Effect of amount offered

**Figure 3:**
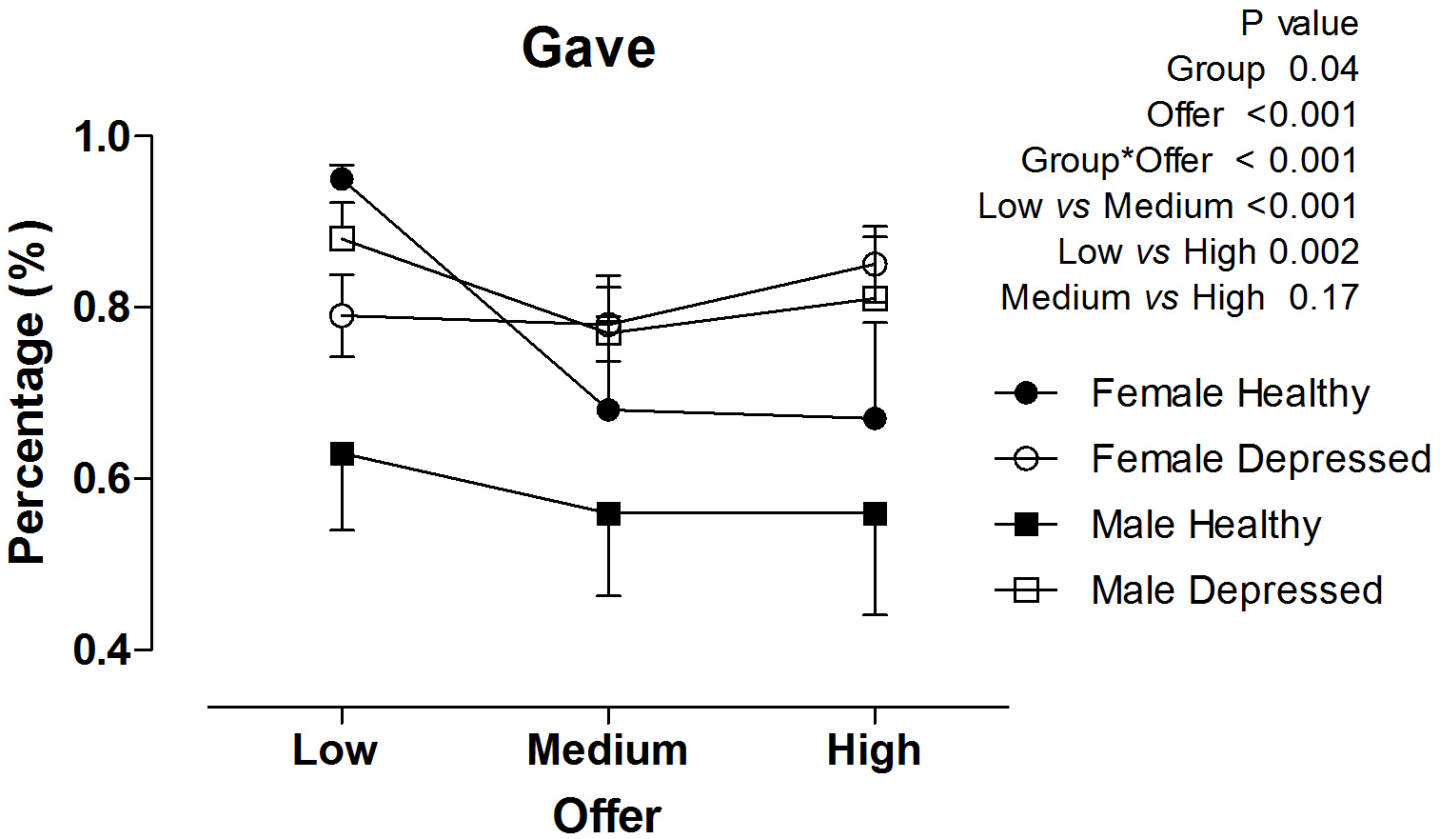
Relationship between offer amount during the baseline condition and reciprocity behavior in healthy women, depressed women, healthy men and depressed men.

In healthy women, the amount of money offered modulated baseline reciprocity behavior, as women were more generous when smaller amounts (<$12) were involved than when larger amounts ($15–$90) were considered (P<0.001, Figure 3). The amount offered had no significant influence in the other studied groups. Healthy men displayed less reciprocity behavior across all offer values compared with the other studied groups (P<0.001, Figure 3).

### Antidepressant effect

Prosocial behavior was not associated with antidepressant therapy, presence of SI, or interaction between these variables.

### Other factors associated with prosocial behavior

To further identify factors influencing behavior in the baseline and the emotionally challenging conditions, we performed linear regressions using either reciprocity or self-centered behavior as dependent variables, and gender, anxiety, emotional abuse, and recent life events as independent variables. Reciprocity behavior correlated positively with anxiety severity (R^2^ = 0.188, P = 0.012), social victimization (R^2^ = 0.082, P = 0.024), and recent social acceptability stress (R^2^ = 0.073, P = 0.033). On the other hand, history of childhood trauma was positively correlated with self-centered behavior (R^2^ = 0.067, P = 0.022).

## Discussion

Our main finding was a gender-specific effect of depression on prosocial behavior: a) healthy men displayed less reciprocity behavior than healthy women; b) depressed men exhibited reciprocity behavior more frequently than healthy men, whereas reciprocity behavior in women did not change with depression; c) depressed women were more self-centered than healthy women; and d) SI induced an inversion of the gender-specific pattern of self-centered behavior: suicidal depressed women were more self-centered, whereas suicidal depressed men were less self-centered.

### Gender differences in prosocial behavior in healthy individuals

Our findings are consistent with previous reports of lower reciprocity in the Trust Game in men compared with women in the general population [5], [28]–[30]. Gender-specific differences in social behavior are mediated by the action of sex hormones and the degree of dimorphic brain structure and function associated with expression of X chromosome-linked genes [31]. For example, estrogen levels are positively correlated with aggression and resentment in women, and progesterone is negatively correlated with suspiciousness and resentment in the luteal phase of the menstrual cycle [32]. Physical violence and sexual aggression are suppressed with estradiol administration [33], [34]. The Ultimatum Game, another monetary exchange task, has provided valuable insight in differential prosocial behavior across genders. In the Ultimatum Game participants are asked to respond to a wide range of offers and reject or punish unsatisfactory offers from another player [35]. Additionally, men with low testosterone are more likely to accept low offers in the Ultimatum Game [36]. Similarly, men with high testosterone levels tend to be more aggressive and display less prosocial behavior [37]. Cooperativeness, a proxy measure of altruism, is higher in women than in men, and is correlated with increased grey matter volume in the posterior inferior frontal and left anterior medial prefrontal cortices [38]. The neural network underlying altruistic behavior has been shown to involve the anterior insular cortex [39] and the amygdala [40] in addition to striatal reward centers [41]. These brain regions have also been shown to be affected in major depression [42], [43]. Hence, it is reasonable to hypothesize that gender differences in prosocial behaviors associated with depression may be related to distinct pattern of neural activation in the limbic system (i.e. insula, amygdala and striatum).

### Comparison with previous studies

We found a gender role reversal in self-centered behavior with depression, but only men showed increased prosocial behavior with depression. To our knowledge gender-specific effects of depression and SI on prosocial behavior have not been previously explored. A recent administration of the Trust Game to adult depressed Chinese women who played the role of the trustee reported less frequency of both altruistic and self-centered behavior than healthy women [44]. However, meaningful comparisons between this study and ours are precluded by major differences in design including a higher number of potential response and outcomes, but mostly a considerable element of risk (the opposing player may find out about the participant’s response) with potential negative consequences to the participant. In contrast, in our study the participant only had the dichotomic choice to reciprocate or not, and he/she was aware that these answers will be known to the other player, but without possibility of payback from the other player. We explicitly followed this design to avoid confounding effects of payback or reputation on the decision whether to reciprocate or not. Increases in prosocial behavior in depressed patients have been demonstrated in a different economic social interaction using the Ultimatum Game. Individuals with depression tended to show strong negative reaction to unfair offers [45], [46]. However, depressed patients tended to offer significantly larger amounts of money than controls [46], [47]. This result is in line with the view of increased prosocial behavior in depressed men as an exaggerated need to accommodate to others, particularly during socially stressful situations, which may reflect an overanxious anticipation of rejection due to inappropriate feelings of guilt and worthlessness [48] and fear of negative evaluation [14]. This explanation is supported by our finding of correlation of increased anxiety and recent social stress with reciprocity behavior. On the other hand, decreased prosocial behavior in depressed women may be associated with negative affect, self-centeredness and decreased ability to care for others.

The explanation for at least part of the gender-specific effect of depression in prosocial behavior could lie in gender-specific abnormalities in guilt and reward processing [49], [50]. Changes in the frequency of engagement in prosocial behaviors may reflect variations in their subjective value compared to that of monetary rewards. Guilt, almost ubiquitously present in severe depression, can be a strong motivator for altruistic behavior [14], [51]. However, further research is warranted to test the role of guilt and anhedonia on the effects of depression on prosocial behavior.

### Gender differences in prosocial behavior in depression

Our study found that depression and SI resulted in reversal of gender differences in prosocial behaviors. The most parsimonious explanation would be that these findings are associated with decreased functional gonadotropin-releasing hormone (GnRH) in depressed suicidal patients [52]. Supporting that connection are studies showing that bioavailable testosterone levels are lower in severely depressed men [53], and administration of GnRH agonist to healthy women is associated with increases in depressive symptoms [54].

Germane to studies of gender-specific behavior and depression, previous reports have found depressed women more often have an anxious and atypical symptom profiles, endorsing more somatic and affective symptoms compared to men [55]–[59]. Depressed women also display a higher tendency to ruminate about problems [60], higher stress, and lower fulfillment associated with traditional gender roles [61].

### Suicidal ideation

There is debate whether suicide represents a severe manifestation of depression or an independent phenomenon. Suicide in the context of mood disorders has been associated with positive and negative moral connotations. Regardless of these differentiations, the reversal of the gender-specific pattern of prosocial behavior in depressed suicidal patients seems to be related to a disruption of HPG axis. For instance, low testosterone levels have been reported in cerebrospinal fluid and serum in suicide attempters [62], [63]. Furthermore, suicide attempters that jumped off heights showed a trend toward lower testosterone levels without increases in luteinizing hormone, suggestive of dysfunction at the hypothalamus-pituitary level [64]. Lastly, suicidal ideation has been associated with cognitive abnormalities including executive function deficits [65] and increased impulsive choice [66]. The potential gender specific role of these cognitive factors in suicidal thinking and their influence on prosocial behavior will need to be further elucidated.

### Possible antidepressants effect

Our results did not show an effect of antidepressant usage on prosocial behavior, although this may be explained by the lack of power since the study was not primarily designed for this purpose. Antidepressants such as selective serotonin reuptake inhibitors (SSRIs), tricyclics, and monoamine oxidase inhibitors are known to induce sexual side effects including decreased libido and anorgasmia. These side effects seem to be GnRH independent [67]; however, antidepressants are associated with increased salivary testosterone in both men and women receiving SSRIs [68]. Further exploration of the effect of antidepressant medication on prosocial behavior is warranted.

### Causality

Even though we did not test for a causal relationship, we believe that the most likely explanation for the observed reversal of gender differences in prosocial behaviors is that depression drives changes in prosocial behavior by modulation of sexual hormones. This hypothesis is based on: a) the association of gender-specific social behavior with sexual hormones in healthy individuals [2]–[4]; b) the hypogonadal state caused by depression via HPA axis hyperactivity and suppression of GnRH secretion [52]; and c) these behavioral changes are more pronounced in the presence of SI, which supports a dose-response relationship with depression severity. However, it is possible that certain coping styles such as masochism [16], [69] lead to pseudoaltruistic behaviors that predispose an individual to the development of mood disorders [17], [70].

### Limitations

In this study we did not use a structured clinical interview, which prevented exploring the role of gender difference in comorbidities including anxiety and personality disorders. Even though we used a widely used behavioral economic tool [71], it is still a simplified model of human social interaction. The interaction in this exchange took place via a computer screen, with no direct personal contact between the players. We did not record income of participants, which has been described as a strong predictor of charitable contributions [72]. Additionally, the majority of the depressed patients were receiving antidepressant pharmacotherapy, and we cannot account for the effect of medication on behavior. Lastly, we did not measure sexual hormones levels.

### Conclusions

We confirmed and expanded gender differences in prosocial behavior in healthy individuals. Depression increased reciprocity behavior in depressed men while increasing self-centered behavior in depressed women. The presence of SI enhanced the effects of depression on self-centered behavior in both genders. Social stress and anxiety in depressed male patients are likely drivers of an exaggerated need to accommodate to others. The pattern of gender-specific changes in social behavior in depressed suicidal patients is suggestive of abnormalities in GnRH secretion and a hypogonadal state. Understanding gender differences in depression may facilitate the design of more efficacious treatments. Further prospective and longitudinal studies are warranted to better confirm our findings, and to relate clinical outcomes to HPA dysfunction.
